# On the Way to the Technological Development of Newly Selected Non-*Saccharomyces* Yeasts Selected as Innovative Biocontrol Agents in Table Grapes

**DOI:** 10.3390/microorganisms12020340

**Published:** 2024-02-06

**Authors:** Antonella Salerno, Margherita D’Amico, Carlo Bergamini, Flavia Angela Maria Maggiolini, Marco Vendemia, Annalisa Prencipe, Claudia Rita Catacchio, Mario Ventura, Maria Francesca Cardone, Antonio Domenico Marsico

**Affiliations:** 1 Council for Agricultural Research and Economics, Research Center Viticulture and Enology (CREA-VE), Via Casamassima 148, 70010 Turi, Italy; antonella.salerno@uniba.it (A.S.); carlo.bergamini@crea.gov.it (C.B.); flavia.maggiolini@crea.gov.it (F.A.M.M.);; 2 Department of Biosciences, Biotechnology and Environment, University of Bari “Aldo Moro”, Via Orabona 4, 70125 Bari, Italyclaudiarita.catacchio@uniba.it (C.R.C.); mario.ventura@uniba.it (M.V.)

**Keywords:** Biological Control Agents, post-harvest decay, table grape, non-*Saccharomyces* yeasts, *Starmerella bacillaris*, *Saturnispora diversa*, *Aureobasidium pullulans*, *Hanseniaspora uvarum*, *Botrytis cinerea*

## Abstract

Post-harvest decay of fresh table grapes causes considerable annual production losses. The main fungal agents of decay both in pre- and post-harvest are *B. cinerea*, *Penicillium* spp., *Aspergillus* spp., *Alternaria* spp., and *Cladosporium* spp. To date, the use of agrochemicals and SO_2_ are the main methods to control grape molds in pre- and postharvest, respectively. Significant improvements, however, have already been made in to apply innovative and more environmentally sustainable control strategies, such as Biological Control Agents (BCAs), which can reduce disease severity in both pre- and post-harvest. In this study, 31 new non-*Saccharomyces* yeast strains, isolated from berries of native Apulian table grape genotypes, were tested for their in vivo effectiveness against grey mold of table grapes, resulting in two *St. bacillaris* (‘N22_I1’ and ‘S13_I3’), one *S. diversa* (‘N22_I3’), one *A. pullulans* (‘OLB_9.1_VL’) and one *H. uvarum* (‘OLB_9.1_BR’) yeast strains that were marked as efficient and good BCAs. Their mechanisms of action were characterized through in vitro assays, and additional characteristics were evaluated to assess the economic feasibility and viability for future technological employment. Their effectiveness was tested by reducing the working concentration, their antagonistic effect on a wide range of fungal pathogens, their ability to survive in formulations with long shelf life, and their safety to human health.

## 1. Introduction

Table grapes are one of the most appreciated fruits by consumers all over the world and, according to the 2022–2023 USDA (United States Department of Agriculture) report, its worldwide production is expected to increase from 1.2 million to 27.4 million tons [1], which represents a 7% year-on-year increase. Table grape production in Europe is also expected to increase from 161,000 to 1.6 million tons in 2023, mainly thanks to a good fruit set in Italy, as well as new seedless varieties going into production in Italy, Spain, and Portugal [1].

However, grape production is annually threatened by serious pre- and post-harvest loss, caused by several rotting agents, among which the most dangerous is *Botrytis cinerea* Pers. Fr. (teleomorph *Botryotinia fuckeliana* (de Bary) Whetzel). This fungus causes grey mold, a disease that every year brings production and financial losses both in fresh grape and wine sectors. In fact, the fungal infection affects the chemical and metabolomic composition of the grape, leading to the deterioration of the grape berries and consequently reducing wine quality [2,3]. Several studies suggest that Quorum Sensing (QS), one of the most studied cell–cell communication mechanisms in fungi, may be behind these infections [4]. Quorum-sensing molecules (QSMs) are known to influence fungal pathogenicity significantly [5], so the study of QS regulation is also important for the treatment of fungal infections. Actually, Quorum Sensing inhibitors (QSIs) of different origins have been shown to act as potential antipathogens [6].

In field conditions, *B. cinerea* develops in favorable weather conditions of high relative humidity and temperatures between 20–25 °C. It can also spread at low temperatures (just above freezing), resulting in infections that can also occur in apparently healthy stored table grapes, without appropriate conditions [7,8]. Under humid conditions, the fungus produces a grey-mold fruiting layer on the affected tissues. The pathogen can cause lesions on the stalk or rachis, leading to premature bunch drop [8]. *B. cinerea* infections in vineyards may start at bloom and remain latent until after veraison, when berry susceptibility to *B. cinerea* rises, because of an increase in sugar concentration and a decrease of antifungal plant compounds. During the ripening period, *B. cinerea* infection is promoted by the presence of micro and macro-wounds on the skin, caused by wind, insects, and compression between berries. In stored table grapes, instead, *B. cinerea* infections generally begins with small necrosis on the skin, which enlarges itself to brown spots. In those areas, the cuticle is separated from the flesh, by macerating enzymes, produced by the fungus. Finally, fungal mycelium starts growing in those areas and grey conidia are originated [9].

Other fungal rot species that can affect table grapes both in field and during storage include *Penicillium*, *Mucor*, *Alternaria*, *Rhizopus*, *Fusarium*, *Aspergillus* [8], *Cladosporium,* and *Aureobasidium* spp. [3]. These fungal pathogens also prove to be very destructive in the commercial distribution stage and at the consumer’s home because they show the ability to grow faster at lower temperatures than *B. cinerea* (10–15 °C) [3].

Chemical treatments at different grapevine phenological stages are normally applied to control grey mold in vineyard conditions; while the use of sulfur dioxide (SO_2_) generator pads is the most common method to preserve the quality and integrity of table grape in post-harvest conditions [7]. In recent years, the development of alternative approaches has been encouraged, aiming to reduce the use of pesticides by 50% before 2030 [10], in order to respond to public concerns regarding the risk of pesticide residues in food, the negative impact of these substances on the environment, and the negative effects that excessive doses of SO_2_ can have both on grapes and human health [11]. An additional reason for reducing the use of synthetic chemical fungicides against fungal rot species is the fast, rapid, and relatively easy selection of resistant strains to single-site fungicides in fungal populations, caused by the continuous use of active substances with the same action mechanisms [12]. Concretely, fungicide resistant strains of grapevine pathogenic molds, such as *B. cinerea* [13], *Penicillium expansum* Link [14] and *Aspergillus* spp. [15], have widely been documented.

Microbial fungicides, based on Biological Control Agents (BCAs), such as bacteria, yeasts, and molds, represent a valid alternative to chemicals for a safer and more effective control strategy [16,17,18,19,20]. Most of the BCAs described against post-harvest pathogens are non-*Saccharomyces* yeasts [21,22,23,24,25], because they offer advantages, such as simple nutritional requirements, the ability to colonize dry surfaces for long periods and the inability to produce allergenic spores or mycotoxins. In 1996, *Rhodotorula glutinis* (Fresen.) F.C. Harrison and *Rhodotorula mucilaginosa* (A. Jörg.) F.C. Harrison were the first yeast species to be patented as BCAs against pathogenic causative agents of grey and blue mold (*Penicillium* spp.), Mucor and secondary fruit rot [26]. In 1998, *Candida oleophila* Kaisha and Iizuka against postharvest diseases caused by *P. expansum*, *Penicillium digitatum* (Pers.) Sacc. and *B. cinerea* [27]. Then, *Metschnikowia fructicola* Kurtzman and Droby was identified as a biocontrol agent against the pathogenic molds *B. cinerea*, *P. digitatum,* and *Aspergillus niger* Tiegh. [28]. Other yeasts that exhibit biocontrol activities against these molds are *Pichia* spp., *Candida guilliermondii* Langeron and Guerra, *Cryptococcus* spp. [29] and *Lachancea thermotolerans* (Filippov) Kurtzman [30]. More recent studies are focused on the presence of grapevine endophytic yeasts belonging to the genera *Metschnikowia*, *Pichia* and *Hanseniaspora* spp. [31,32]. In this work, we screened the effectiveness of native vineyards non-*Saccharomyces* yeasts against *B. cinerea*. A stepwise screening program divided into six phases was followed: (i) selection of the most suitable niches; (ii) isolation of microorganisms; (iii) preliminary assessments by rapid screening assays; (iv) identification of candidate antagonists; (v) study of their mechanism of action; (vi) evaluation as ideal biocontrol agents. In vivo experiments were performed to select BCAs candidates among thirty-one non-*Saccharomyces* yeasts, isolated from the carposphere from new *V. vinifera* selected genotypes, obtained in the Breeding Program of Council for Agricultural Research and Economics—Research Center Viticulture and Enology (CREA-VE, Turi, Southern Italy). Furthermore, as understanding the mode of action is essential to developing an appropriate formulation and methods of application and to obtain registration [16], a preliminary characterization of the mechanism of activity was performed, through different in vitro assays. Finally, the best-performative yeasts were characterized for some of the ideal biocontrol agents features, enumerated by Droby et al. [33], such as (i) effectiveness at low concentrations, (ii) efficacy against a wide range of pathogens, (iii) amenability to formulation with long shelf-life and (iv) not showing adverse effects to human health.

## 2. Materials and Methods

### 2.1. Yeast Isolation and Culture Conditions

In total, 31 Non-*Saccharomyces* yeasts used in this study were isolated from the grape berries of seven new *V. vinifera* genotypes, obtained through the CREA-Research Center Viticulture and Enology (CREA-VE) breeding program. All the genotypes are cultivated in an experimental vineyard, located in Rutigliano (BA) (40°57′26.65″ N, 17°00′31.34″ E, 185 mt. a.s.l.), with their selection based on their high bunch compactness and different degree of tolerance to grey mold infections (Table S1).

Apparently healthy berries sampled from each genotype were placed in a full-page microperforated-filter blander bag (filter porosity = 63 microns) (Bag Filter^®^—Interscience Bag System^®^, Saint-Nom-la-Bretèche, France) and manually crushed to obtain grape juice ready for the isolation protocol. Appropriate dilutions of grape juice were aseptically plated on Wallerstein Laboratory Nutrient Medium (WLN (VWR Chemicals, Leuven, Belgium)) amended with chloramphenicol (Sigma-Aldrich, St. Louis, MO, USA) 50 mg L^−1^ to avoid bacterial growth. Plates were incubated for three days at 25 °C. Well-developed yeast colonies were grouped, based on their color and morphology [34]. Representative colonies for each group were selected and then transferred to new WLN plates. Finally, pure colonies were grown on liquid Yeast Extract Peptone Dextrose (YPD: 1% yeast extract (VWR Chemicals, Leuven, Belgium), 2% peptone (VWR Chemicals, Leuven, Belgium), 2% dextrose (VWR Chemicals, Leuven, Belgium)) for 48 h at 25 °C and then stored at −80 °C in liquid YPD with 30% (*v*/*v*) of glycerol (Carlo Erba Reagents, Val-de-Reuil, France).

### 2.2. Fungal Pathogens Isolation, Pathogenicity Test and Culture Conditions

Fungal pathogens were isolated from different vegetal matrices (Table S2) and all of them were subjected to a two-step purification procedure on Water Agar (WA) in order to obtain monohyphal cultures [35]. To allow the pure colony growth of the pathogen, the purified hyphal portion was then transferred with a sterile needle onto Potato Dextrose Agar (PDA) (VWR Chemicals, Leuven, Belgium) and finally stored in a glass tube containing Agar Potato Sucrose (APS) closed with a cotton cap. The virulence of each fungal isolate was tested in “Italia” wounded grape berries. Twenty-four mature berries for each fungal isolates were disinfected for 5 min in a solution of sodium hypochlorite (3.5% active chlorine), rinsed in sterile water and air-dried [30]. Wounds were made on the berries with a sterile scalpel and inoculated with a 20 μL drop of 1 × 10^5^ conidia mL^−1^ of the fungal isolate. The berries were incubated at 25 °C in sterile plastic boxes in a humid chamber. The ability of each fungal isolate to develop the symptoms of the disease was evaluated after five days.

### 2.3. Preliminary Screening for Antagonistic Activity

The antagonistic activity of 31 non-*Saccharomyces* yeast isolates against *B. cinerea* was preliminarily evaluated by performing two consecutive in vivo experiments. In both assays, mature “Red Globe” grape berries were collected from healthy bunches, preserving their pedicels. Their surface was sterilized by dipping in sodium hypochlorite (3.5% active chlorine) solution for 5 min, washed in sterile water two times and then air dried. Artificial wounds were performed along the berry equatorial area. Thirty grape berries for each yeast isolate were placed in three plastic boxes and each wound was inoculated with 20 µL drop of yeast cells suspension at the concentration of 1.5 × 10^7^ CFU mL^−1^. Thirty grape berries inoculated with 20 µL of sterile water were used as control. After 48 h of incubation at 25 °C each wound was inoculated with 20 µL of a conidia suspension of *B. cinerea* at the concentration of 1 × 10^5^ conidia mL^−1^. The Disease Severity (DS) was evaluated five days after pathogen inoculation and incubation at 25 °C by using an empirical 0- to –4 rating scale, in which 0 = no visible symptoms; 1 = sporulation covering 5–10% of the wound surface; 2 = sporulation covering 10–25% of the wound surface; 3 = sporulation covering 25–50% of the wound surface; 4 = sporulation covering more than 50% of the wound surface. The average disease severity was calculated for each plastic box by using McKinney’s formula [36] and the effectiveness (%) of each yeast strain to control disease severity was calculated using the following formula [37]:

E(%) = (1 − T1/C1) × 100(1)

where:
T1 = the average grey mold severity detected in treated grape berries
C1 = the average grey mold severity detected in untreated grape berries

### 2.4. Molecular Identification of Selected Yeast and Pathogenic Molds

Yeast and molds DNA extraction was performed with the FastDNA^®^ SPIN kit for soil (MP Biomedicals, LLC, Solon, OH, USA). Pure cultures of yeast were selected in order to avoid intraspecies diversity, they were grown in liquid YPD and centrifuged for 20 min at 4000 rpm and then the pellet was processed following manufacturing protocol. DNA concentration and purity were evaluated spectrophotometrically.

Molecular identification of yeasts selected as effective BCAs and of pathogenic molds used in the experiments was carried out by Illumina technology (Illumina Hayward, Hayward, CA, USA). The nuclear ribosomal internal transcribed spacer 2 (ITS2) region of the fungal DNA was amplified using a mono-index approach following the protocol reported by Taylor et al. [38] [NO_PRINTED_FORM]. PCR amplification was carried out in a final volume of 20 μL, including 20 ng of DNA template, 4 µL of HOT FIREPol^®^ MultiPlex Mix (Solis BioDyne OÜ, Tartu, MA, Estonia), 1.25 μL of each primer 10 µM, and 1.5 μL of grape ITS blocking primer 10 µM (sequence: CGAGGGCACGCCTGCCTGG). Finally, the pooled PCR products were size-selected with an Invitrogen^®^ (Thermo Fisher Scientifics, Waltham, MA, USA) 2% E-Gel, and purified using the QIAquick purification kit (Qiagen, Germantown, MD, USA). Their concentration was determined with the Qubit^®^ dsDNA HS Assay Kit (Thermo Fisher Scientifics, Waltham MA, USA). The pooled PCR products were sequenced with an Illumina^®^ MiSeq sequencer (2 × 300 cycles) (Illumina, San Diego, CA, USA). 25% PhiX control DNA was spiked in the run to add base diversity. Data from Mi-Seq sequencing were analyzed; each sample’s reads were multialigned using MEGA11 Software and the consensus sequence was obtained by Waterhouse et al. [39]. The species identification was carried out by scanning the Fungi/Metazoa group (taxid 33154) from NCBI blastn suite (https://blast.ncbi.nlm.nih.gov/Blast.cgi?PAGE=MegaBlast&PROGRAM=blastn&BLAST_PRO-GRAMS=megaBlast&PAGE_TYPE=BlastSearch&DBSEARCH=true&QUERY=&SUBJECTS=, accessed on: 1 December 2023) through blast-search, as previously reported [40,41,42,43].

When the Illumina sequencing identity percentage was too low, the molecular identi-fication of yeasts was confirmed using the Sanger method by sequencing the D1/D2 26S DNA using the primers NL1 and NL4 [40,41], while the molecular identification of molds was confirmed by the Sanger method by sequencing the 5.8SDNA [44,45] of each isolate with the primers ITS1 and ITS4 [40,41]. PCR amplification was carried out in a final volume of 15 μL, including 10 ng of DNA template, 1× buffer, MgCl_2_ 1.6 μM, dNTPs 200 μM, primers) 0.5 μM, Taq pol 1.5U. PCR products were purified using the PCR clean-up and Gel extraction purification kit (MACHEREY-NAGEL, Düren, Germany). 

### 2.5. Characterization of the Mechanism of Action

Three different in vitro assays were performed to obtain further information regarding the mechanisms of action of five selected non-Saccharomyces yeast strains. In particular, the first one was carried out with the Cellophane Agar Layer (CALt) technique [46] to evaluate the yeasts’ ability to produce fungistatic diffusible substances; the second one with the sandwich dual culture technique [47], to evaluate the ability to produce fungistatic Volatile Organic Compounds (VOCs). Both experiments were performed as reported by Marsi-co et al. 2021 [30]. Finally, based on the results collected in the previous two experiments, three non-Saccharomyces yeast strains were selected and evaluated for their ability to pro-duce lytic enzymes (lipase, esterase, β-1,3-glucanase, chitinase, protease and pectinase) by streaking each yeast strains onto specific grow media, both in presence and absence of *B. cinerea*. (I) Lipase activity was evaluated on tributyrin agar medium (pH = 6) [48]; after the incubation for five days at 25 °C, a clearer zone around the yeast colonies expressed the lipase activity. (II) Esterase activity was also tested following the indications of Buzzini and Martini, (2002) [48], using a solid medium (pH = 6.8) containing 10 g L^−1^ of TWEEN 80 (VWR Chemicals, Leuven, Belgium), 10 g L^−1^ of peptone (VWR Chemicals, Leuven, Belgium), 5 g L^−1^ of NaCl (Sigma-Aldrich, St. Louis, MO, USA), 0.1 g L^−1^ of CaCl_2_∙2H_2_O (Sigma-Aldrich, St. Louis, MO, USA) and 6.8 g L^−1^ of Agar (VWR Chemicals, Leuven, Belgium) [49]; after the incubation periods of five days at 25 °C, a clearer zone around the isolates determined the esterase activity [50]. (III) β-1,3-glucanase solid medium (pH = 7) was prepared using 5.0 g L^−1^ of glucan (Tokyo Chemical Industry, Tokyo, Japan), 6.7 g L^−1^ of Yeast Nitrogen Base (YNB) (VWR Chemicals, Solon, Ohio, USA) and 15.0 g L^−1^ of Agar; after the incubation period of 72 h at 25 °C the plates were covered with 0.6 g L^−1^ of Congo Red (Sigma-Aldrich, St. Louis, MO, USA) and left to rest at 25 °C for 90 min; once removed the excess dye, the capacity to hydrolyse glucan was evaluated by assessing a yellow-orange zone around the colonies [51]. (IV) Chitinase solid medium was prepared following the second method reported by Roberts and Selitrennikoff (1988) for the addition of colloidal chitin [52], and the method of Souza et al. (2009) for the mineral salts [53]; detection of extracellular chitinase activity was assessed after an incubation period of seven days at 25 °C by the observation of a clearer zone around the inoculum zone. (V) Protease activity was evaluated following Strauss et al. (2001) indications: YPDA was amended with 20 g L^−1^ of casein (Merk KGaA, Darmstadt, Germany) (pH = 7) and left to incubate for seven days at 25 °C; a clearer zone around the isolates expressed the ability to degrade casein [54]. (VI) Pectinase activity was evaluated on solid medium (pH = 7) containing 10.0 g L^−1^ of citrus pectin (Thermo Fisher, Kendel, Germany), 6.7 g L^−1^ of YNB (VWR Chemicals, Solon, Ohio, USA) and 15 g L^−1^ of Agar; after 72 h of incubation at 25 °C, the plates were flooded with 10.0 g L^−1^ of hexadecyltrimethylammonium bromide (Alfa Aesar GmbH & Co., Karlsruhe, Germany): degradation of pectines was evaluated through observation of a clearer zone around the colonies [48]. For each combination yeast strain/growth media, six replicates (plates) were realized: three plates in which the yeast alone was streaked to form a square in the center of the plate, and three plates in which a 9 mm mycelial disc of *B. cinerea* was placed in the center of the square formed with the yeast streak. 

### 2.6. Characterization of More Ideal Features of a BCA

Yeast isolates ‘N22_I1’, ‘S13_I6’, ‘OLB_9.1_VL’, ‘N22_I3’ and ‘OLB_9_BR’, selected as effective BCAs against *B. cinerea*, were further analyzed for some of the characteristics that an ideal biocontrol agent should have as enumerated by Droby et al. [33] (effectiveness at low concentrations and against a wide range of pathogens, amenable to formulations with long shelf-life and their safety to human health). (I) Effectiveness of selected yeast isolates when applied at low concentrations (1.5 × 10^5^ CFU mL^−1^) to control Botrytis bunch rot, Aspergillus black mold, Penicillium green and blue mold, Cladosporium brown spot and Alternaria decay, was evaluated in the wounded berries assays, performed as described in Section 2.3. (II) To evaluate their ability to survive after a freeze-drying process, yeast isolates were cultivated in four falcon tubes, containing 5.0 mL of liquid YPD each. After 24 h (T0), one falcon tube for each yeast isolate was used to evaluate the number of viable cells before the freeze-drying. The remaining three falcon tubes for each yeast strains were centrifuged at 4000 rpm for 20 min, with the supernatant then removed. The pellets were freeze-dried in a LIO5P 4K lyophilizer (Cinquepascal s.r.l., Milano, Italy); after the procedure the tubes were sealed before vacuum application. One falcon tube for each yeast isolates was immediately used to evaluate the number of viable cells after the freeze-drying (T1), while the other two were stored both at room temperature and at 4 °C for 30 days (T2_25° and T2_4°, respectively). Viable cell counts at each time points were carried out on WL plates; in details, at T0, 1 mL was serially diluted and spread on plates; differently at T1 and T2 the lyophilized yeast was prior resuspended in 1 mL of liquid YPD before being serially diluted and spread on the plates. Plates were incubated at 25 °C for 3–4 days and then the number of Colony-Forming Units per milliliter (CFU mL^−1^) was determined. A Survival Factor in the Lyophilization process (SFL) was defined as follows [55]:

SFL = 1 − [(log CFU/mL T0 − log CFU/mL T1)/log CFU/mL T0](2)

where:
CFU/mL T0: number of viable cells before the lyophilization process (T0)
CFU/mL T1 number of viable cells after the lyophilization process (T1)

After the storage at both temperatures, the number of CFU mL^−1^ was used to calculate a Survival Factor to Storage (SFS) according to the following equation:

SFS = 1 − [(log CFU/mL T1 − log CFU/mL T2)/log CFU/mL T1] (3)

where:
CFU/mL T1 number of viable cells after the lyophilization process (T1)
CFU/mL T2 number of viable cells after the storage (T2_4° or T2_25°)

(III) Selected yeast isolates were also tested for their ability to produce haemolysin and a consequently possible, deleterious action on human red blood cells (erythrocytes) [56]. The experiment involved a plate assay system where yeasts were incubated on dextrose (1%)-enriched sheep blood agar 5% (*v*/*v*) (VWR BDH CHEMICALS—Blood Agar Base—84,619.0500) [56,57]. A 20 µL solution of each yeast was plated by a zig-zag streak [58] on a dextrose (1%)-enriched blood agar plate with a sterile loop. Six replicates for each isolate were set up. Three plates were incubated at 25 °C (optimal temperature for yeast growth) and three plates were incubated at 37 °C (human body temperature). After five days of incubation, β-haemolysis was observed by a clear zone around the yeast colony, indicating erythrocyte breakage; α-haemolysis or partial haemolysis was represented by a color change to dark-green, indicating a reduction of red blood cells’ haemoglobin to methae-moglobin. Non-alteration over the medium (γ-haemolysis) indicates no damage to erythrocytes [59].

### 2.7. Statistical Analysis

The data were analyzed using RStudio software (v. 4.2.3). Data normality, homoscedasticity and homogeneity of variances were evaluated by Shapiro–Wilk’s test, Bartlett’s test and Levene’s test, respectively. When at least one of the three conditions was satisfied, we performed the analysis of variance using the parametric ANOVA test (*p* < 0.05), followed by Turkey’s post hoc test.

## 3. Results

**Figure 1:**
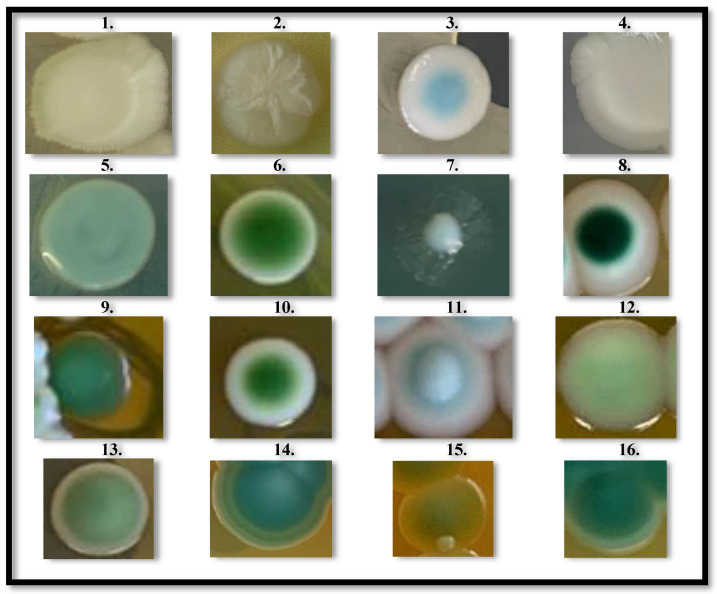
Yeasts colonies morphology. A total of 16 different morphologies of isolated yeast colonies, distinguished by color and topography: (1) Cream to light green/yellowish medium, Flat, surface: smooth/opaque; (2) White to cream/yellow medium, Convex, surface: wrinkled/opaque; (3) Light blue in the center/cream at periphery, Flat, surface: smooth/glossy; (4) White to cream/yellowish medium, Flat, surface: smooth/opaque; (5) Light green/yellowish medium, Flat, surface: smooth/glossy; (6) Intense green in the center/cream at periphery, Flat, surface: smooth/glossy; (7) Light green with thin hyphal-like ramifications/yellowish medium, Convex, surface: smooth/glossy; (8) Dark green in the center/white at periphery, Flat, surface: smooth/glossy; (9) Intense green/greenish medium, Convex, surface: smooth/opaque; (10) Intense green in the center/white at periphery, Convex, surface: smooth/glossy; (11) Intense white in the center/light green at periphery, Convex, surface: smooth/glossy; (12) Light green/yellowish medium, Flat, surface: smooth/glossy; (13) Intense green in the center/light green at periphery/yellowish medium, Convex, surface: smooth/glossy; (14) Light blue in the center/intense blue in the periphery, Convex, surface: smooth/opaque; (15) Light green tending to yellow/yellow medium, Flat, surface: smooth/opaque; (16) Intense green with light green edge/yellowish medium, Convex, surface: smooth/opaque.

From the grape juice obtained from seven selected new *V. vinifera* genotypes (Table S1), characterized by having compact bunches and a constant response to *B. cinerea* infection as noted through many years of observation, several yeast colonies were collected and divided into 16 different groups based on the morphotype expressed on WL Nutrient agar. From this preliminary discrimination (Figure 1), a random selection of 1–3 yeast colonies from each of the 16 groups resulted in a total of 31 yeast strains (Table S3), that would have expanded the CREA-VE yeast collection and further studied in this work.

### 3.1. Fungal Pathogens Isolation and Pathogenicity Tests

Molds used for the following antagonism experiments were isolated from different vegetable matrices. The molds species identity was assessed through morphological analysis and then confirmed by Illumina sequencing (Table S2). Data revealed that molds isolates belonged to the following species: *B. cinerea* (AS1) with a sequencing match of 94%, *Alternaria alternata* (Fr.) Keissl. (AS9), with 45% sequencing match, *P. digitatum* (AS13), with 94% sequencing match of, and *A. niger* (AS19) with a sequencing match of 68% (Table S2). Illumina sequencing matches for the isolates AS3 (*Cladosporium* sp.) and AS14 (*Penicillium glabrum* (Wehmer) Westling) was lower than 40% (36 and 34%, respectively); for those molds Sanger sequencing was performed to confirm the molecular identification of genera and/or species (Supplementary Material D1.1). Moreover, when species identification for the AS3 isolate could not be confirmed by the Sanger sequencing, the 5.8S sequence obtained from the AS14 isolate aligned to the ITS fungal database and showed 99.40% identity with the *P. glabrum* species, in agreement with the Illumina sequencing (Table S3). The latter result was also supported by acknowledging *P. glabrum* as the most common yeast strain diffused in Southern Italy [60,61]. The pathogenic ability to induce infection on sterile grape berries of the cultivar “Italia” was tested for all isolates. All six fungal isolates were able to develop disease symptoms on the berries after a 5-day incubation at 25 °C (Figure S1).

### 3.2. Preliminary Screening for Antagonistic Activity

**Figure 2:**
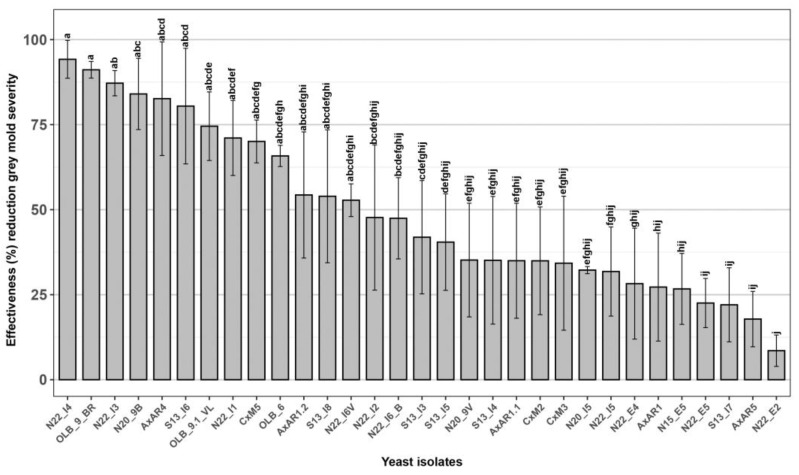
Effectiveness of 31 yeast isolates against *Botrytis cinerea* bunch rot. In vivo antagonistic activity of 31 yeast isolates to inhibit grey mold decay on wounded grape berries. Data are presented as a percentage reduction of disease severity (McKinney Index) compared to the untreated control. The columns labeled with different letters are statistically significant according to Tukey’s test (*p* < 0.05).

All the 31 new yeasts isolated from grape bunches of 7 native new table grape genotypes were tested through in vivo experiments to evaluate their effectiveness against the bunch rot caused by *B. cinerea*, assessed as a percentage reduction of disease severity, compared to the untreated control [36,62]. Among the 31 yeasts strains only 10, named ‘N22_I4’, ‘OLB_9_BR’, ‘N22_I3’, ‘N20_9B’, ‘AxAR4’, ‘S13_I6’, ‘OLB_9.1_VL’, ‘N22_I1’, ‘CxM5’ and ‘OLB_6’, showed an effectiveness greater than 60.0% and therefore selected for the subsequent analysis (Figure 2).

**Figure 3:**
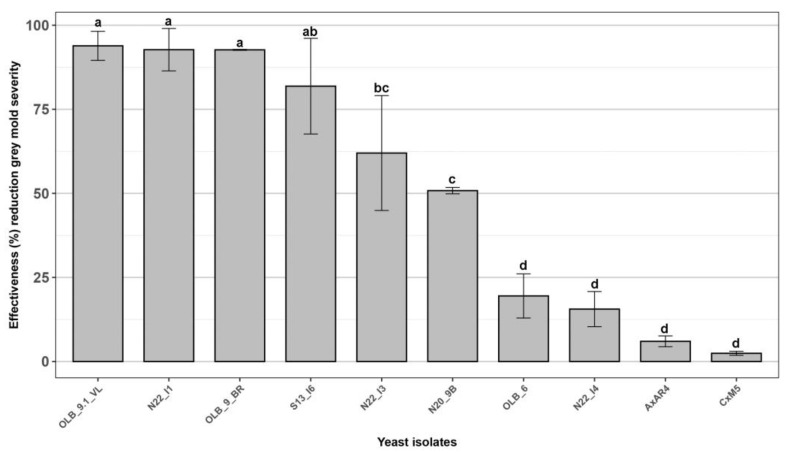
Effectiveness of ten yeast isolates against *Botrytis cinerea* bunch rot. In vivo antagonistic activity of ten yeast isolates in inhibiting grey mold decay on wounded grape berries. Data are presented as a percentage reduction of disease severity (McKinney Index) compared to the untreated control. The columns labeled with different letters are statistically significant according to Tukey’s test (*p* < 0.05).

The ten most effective yeast isolates, selected in the first preliminary tests, were used to perform a further in vivo antagonism assessment aimed to confirm their effectiveness against grey mold of table grapes. An efficacy greater than 60.0% was confirmed for the five yeast strains ‘OLB_9.1_VL’, ‘N22_I1’, ‘OLB_9_BR’, ‘S13_I6’ and ‘N22_I3’ (Figure 3), that were then selected for further characterization studies.

### 3.3. Morphological and Molecular Identification of Selected Yeasts

The morphological characteristics of the five selected yeasts are reported in Table S4 and include the color and morphology of the pure colonies on the WL medium. Moreover, to more precisely assess these five yeast species we performed Illumina sequencing on DNA extracted from the purified colonies and identified through BLAST search. Due to the high yeast genetic diversity, difficulties in the characterization of species have been previously reported; however, the expansion of the GenBank sequences repository makes BLAST identification the more suitable method used for yeast characterization [40,41,42,43]. The analysis identified two *Starmerella bacillaris* (Kroemer & Krumbholz) F.L. Duarte & Á. Fonseca (‘N22_I1’ and ‘S13_I6’), one *Hanseniaspora uvarum* (Niehaus) Shehata, Mrak & Phaff (‘OLB_9_BR’) with a species match greater than 89% and one *Aureobasidium pullu-lans* (de Bary & Löwenthal) G. Arnaud (‘OLB_9.1_VL’), with a species match of 70%. Additionally, the yeast isolate ‘N22_I3’ was identified by Illumina sequencing as *Saturnispora diversa* (Ohara, Nonom. & Yunome ex van Uden & Buckley) Kurtzman with a match lower than 40%, thus, Sanger sequencing needed to be performed to confirm the species identity (Supplementary Material D1.2). The alignment of the ‘N22_I3’ isolate’s consensus sequence against the ITS fungal database showed 99% identity with the species *S. diversa*, confirming the Illumina sequencing results. Anyway, a more precise taxonomic identification would be performed for those isolates showing the best performances as candidate BCA.

### 3.4. In Vitro Tests to Characterize the Antagonism Mechanism of the Non-Saccharomyces Yeasts

**Figure 4:**
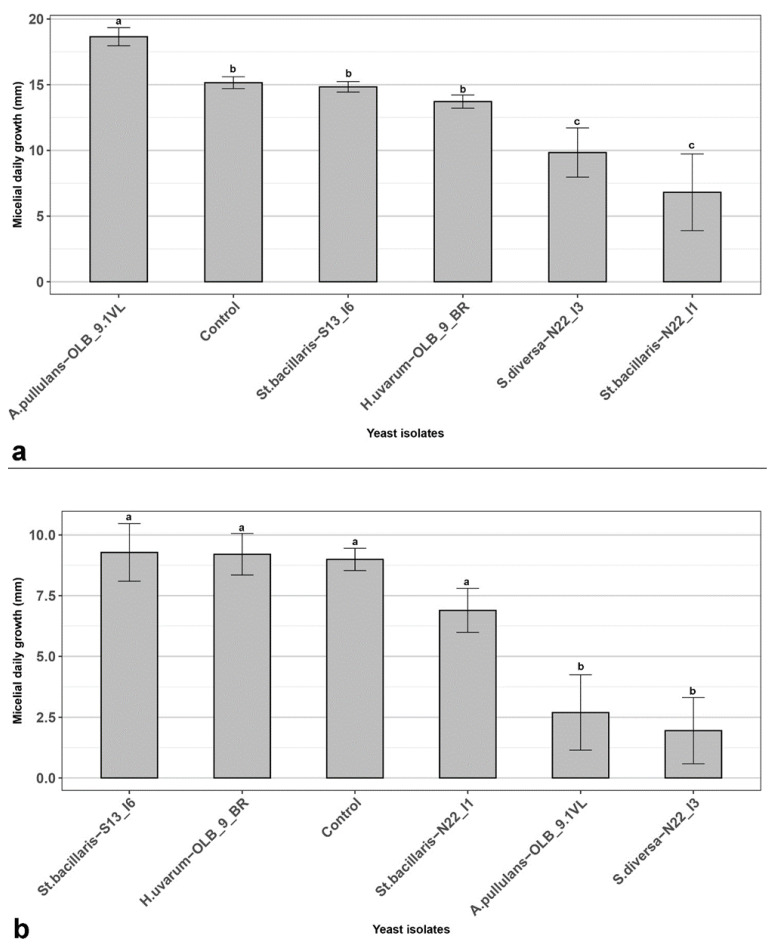
In vitro assays (**a**) Cellophane agar layer technique (CALt); (**b**) Sandwich dual culture for Volatile Organic Compounds (VOCs). In vitro antagonistic activity of yeast strains refers to mycelium daily growth of *Botrytis cinerea*. Plates without yeast strains were used as controls. Data are presented as the mean of five replicates with standard deviation (vertical bars). Columns labeled with different letters are statistically significant according to Tukey’s test (*p* < 0.05).

**Figure 5:**
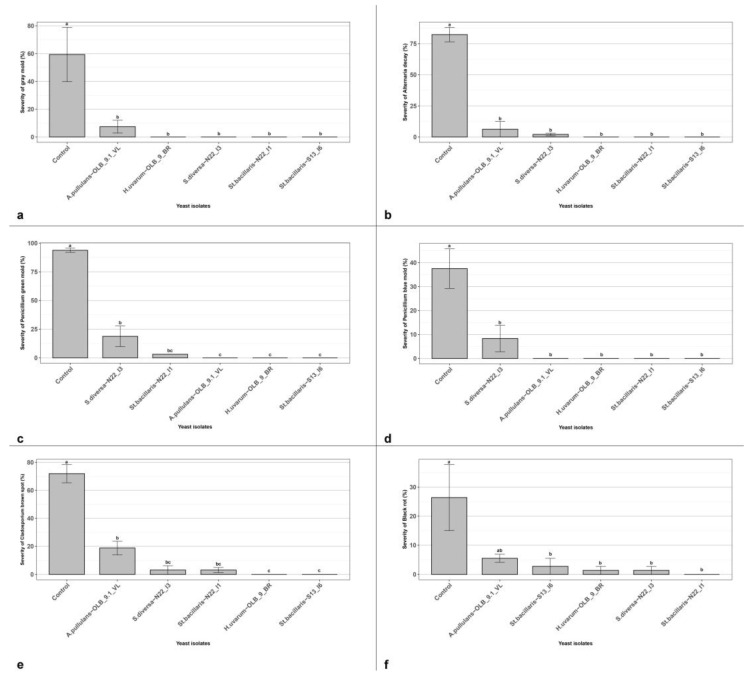
Yeast efficacy at lower concentration (1.5 × 10^5^ CFU mL^−1^). In vivo antagonistic activity of five yeast isolates to inhibit grey mold decay, caused by *B. cinerea* (**a**), Alternaria decay, caused by *A. alternata* (**b**), Penicillium green mold, caused by *P. digitatum* (**c**) Penicillium blue mold, caused by *P. glabrum* (**d**), Cladosporium brown spot, caused by *Cladosporium* spp. (**e**) and black rot, caused by *A. niger* (**f**) on wounded grape berries. Data are presented as the mean of five replicates with standard error (vertical bar). Columns labeled by different letters are significantly different according to Tukey’s test (*p* < 0.05).

To characterize the mechanism of action of the selected yeasts throughout the preliminary experiments, three in vitro experiments were performed. In the first two, their possible ability to reduce *B. cinerea* mycelium growth via the production of diffusible substances [46] (Figure 4) and/or VOCs [47] (Figure 5) were evaluated by using a yeast suspension at 1.5 × 10^7^ CFU mL^−1^, evaluating the mycelium growth reduction of *B. cinerea* as its daily percentage reduction in the presence of the antagonist yeast isolates, compared to the control without yeasts [30]. Results in CALt showed that, the *S. diversa* strain ‘N22_I3’ and *St. bacillaris* strain ‘N22_I1’ were able to significantly reduce the daily mycelium growth of *B. cinerea* (Figure 4a). Similarly, the *S. diversa* strain ‘N22_I3’ and *A. pullulans* strain ‘OLB_9.1_VL’ showed a significant ability to reduce the mycelium growth of the pathogen thanks to VOCs activity (Figure 4b). In detail, the *S. diversa* ‘N22_I3’ significantly reduced the in vitro growth of the fungus in both experiments by 35.1 and 80.1%, respectively. On the other hand, the *St. bacillaris* ‘N22_I1’ significantly reduced the mycelium growth of *B. cinerea* in CALt experiment by 55.1%, while *A. pullulans* ‘OLB_9.1_VL’ significantly reduced the mycelium growth of the fungus in VOCs experiment by 69.4%. *St. bacillaris* strain ‘S13_I6’ and *H. uvarum* strain ‘OLB_9_BR’ did not significantly reduce the daily growth of *B. cinerea* in both experiments.

To further characterize the mode of action of diffusible or volatile substances produced by *St. bacillaris* strain ‘N22_I1’, *S. diversa* ‘N22_I3’ and *A. pullulans* ‘OLB_9.1_VL’, their specific enzymatic activity was investigated. The ability of yeasts to produce lytic enzymes was tested, using different selective substrates both in presence (P.) and in absence of the pathogen (W.P.). Results reported in Table 1 showed that *A. pullulans* ‘OLB_9.1_VL’ and *S. diversa* ‘N22_I3’ yeasts strains were able to hydrolase tributyrin (lipase activity) only when they were in the presence of the pathogen. Additionally, *A. pullulans* ‘OLB_9.1_VL’ showed protease activity in the presence of the pathogen and esterase activity when not in contact with the pathogen. Finally, the *St. bacillaris* strain ‘N22_I1’ was unable to grow in specific growth media to assess lipase, esterase, β-1.3-glucanase, and chitinase activity, while not showing protease and pectinase activity.

### 3.5. Characteristics Evaluation of Ideal Biocontrol Agents

The five isolates being evaluated as effective biocontrol agents of *B. cinerea (St. bacillaris* ‘N22_I1’ and ‘S13_I6’, *S. diversa* ‘N22_I3’, *H. uvarum* ‘OLB_9_BR’, and *A. pullulans* ‘OLB_9.1_VL’) were further characterized for other characteristics considered ideal for biocontrol agents as described by Droby et al. [33]. We evaluated their effectiveness at low concentrations, both against *B. cinerea* and a wide range of pathogens, their ability to survive in formulation with long shelf life, and their safety to human health.

#### 3.5.1. Effectiveness at Low Concentrations and Against a Wide Range of Pathogens

We tested the isolate ability to be effective even when applied at lower concentrations of 1.5 × 10^5^ CFU mL^−1^ against *B. cinerea* and other secondary rot agents, such as *A. niger, A. alternata, P. glabrum*, *P. digitatum and Cladosporium* spp. All the tested yeast strains confirmed their ability to significantly reduce the grey mold severity, even when applied at the concentration of 1.5 × 10^5^ CFU mL^−1^. All the yeast strains significantly reduced the disease symptoms from a minimum of 91.30% (*A. pullulans* strains ‘OLB_9.1_VL’) to a maximum of 100% (all others) (Figure 5a). In the in vivo conditions realized in this experiment, the five yeast strains showed a different ability to reduce the disease severity caused by other secondary rot agents (Figure 5). All yeast isolates resulted effective against four over five tested secondary agents; and *St. bacillaris*, the *S. diversa* and the *H. uvarum* were able to significantly reduce the disease severity of black rot, caused by *A. niger*.

#### 3.5.2. Freeze Drying Process

The resistance of selected yeast strains to the lyophilization process (Survival Factor to the Lyophilization—SFL) and their viability when stored at different temperatures (4° and 25 °C) (Survival Factor to Storage—SFS) were evaluated. All the selected yeast strains showed high SFL, close to 1.0 and no significant differences were observed between strains. A full two-factor ANOVA performed using data collected during the storage of freeze-dried microorganisms showed that the different survival degrees during storage (SS = 2.80, *p* < 0.0001) among yeast strains were significantly affected by the storage temperatures (SS = 0.25, *p* = 0.015). As reported in Table 2, the *St. bacillaris* ‘N22_I1 and *S. diversa* ‘N22_I3 showed higher SFS at both refrigerated (0.85 ± 0.01 and 0.70 ± 0.01, respectively) and room temperature (0.90 ± 0.02 and 0.78 ± 0.01, respectively). The *A. pullulans* strain OLB_9_VL and *St. bacillaris* strain ‘S13_I6 only showed high SFS when stored at 4 °C (0.67 ± 0.003 and 0.78 ± 0.002, respectively); differently, when the freeze-dried powder was stored at room temperature, SFS was significantly reduced for *A. pullulans* strain OLB_9_VL (SFs = 0.47 ± 0.003) and reached values equal to 0.00 for *St. bacillaris* strain ‘S13_I6. Finally, regarding *H. uvarum* ‘OLB_9_BR’, the storage at both refrigerated and room temperature negatively affected their survival.

#### 3.5.3. Yeasts Haemolytic Activity

The previous five yeast strains were investigated for their ability to lyse the red blood cell membrane through haemolysin production. As shown in Table 3, none of the yeasts can grow on blood agar at the human body temperature (37 °C), and only two yeasts (*S. diversa* N22_I3 and *A. pullulans* OLB_9.1_VL) grow at the temperature of 25 °C. In particular, no clear or brown areas were detected around the colony of *S. diversa* N22_I3, suggesting the inability of this yeast to produce haemolysin (γ-haemolysis). Differently, a clear area was detected around the colonies of *A. pullulans* OLB_9.1_VL grown on blood agar and this highlights the ability of the yeast strain to degrade the red blood cell membrane (β-haemolysis).

## 4. Discussion

One of the most important problems in table grape production is the deterioration of berries during storage, transportation, and marketing before the product reach consumers’ tables [16]. Currently, the most widespread strategy to preserve table grapes after harvest is the use of pads releasing SO_2_ [63,64]. Even though this compound is registered as an adjuvant in most countries, it was removed from the “Generally Recognized as Safe” (GRAS) list, classified as pesticide in the USA and it is not allowed on organic grapes [65]. These issues led to an enhanced interest in new alternative strategies, which include the realization of bio-fungicides based on antagonistic microorganisms [66]. The development of a BCA for pre- and/or post-harvest disease is a long, costly, and interactive process that involves several steps, among which the choices made in the isolation step strongly in-fluence the success of the selected microorganism under commercial conditions. In our study, we recovered 31 yeasts isolated from the carposphere of seven new table grape gen-otypes characterized by high bunch compactness (a physical feature predisposing grey mold infections) and different degrees of tolerance/susceptibility to grey mold. Notably, 13 yeast strains (about 65.0%) were isolated from highly tolerant genotypes, six strains (about 30.0%) from mediumly tolerant genotypes and only one (about 5.0%) from highly susceptible genotypes. In addition, results of the antagonistic screening performed by in vivo assays demonstrated that five (*St. bacillaris* ‘N22_I1’ and ‘S13_I6’, *S. diversa* ‘N22_I3’, *H. uvarum* ‘OLB_9_BR’, *A. pullulans* ‘OLB_9.1_VL’) of the 31 new yeast strains (about 16.0%) were able to inhibit grey mold by 60.0% or more. Nunes et al. [67] tested in ‘Blanquilla’ pears the activity of 247 bacteria and yeasts, isolated from the fruit and leaf surface, against *P. expansum* showing that only the 2.0% inhibited decay by 50.0% or more. In another study, among 1440 microorganisms isolated from the surface of leaves of orange trees only four (about 0.5%) of them showed a potential role in being labeled as BCAs against green mold in oranges [68]. These contrasting results might be related to the adopted selection protocol, as isolating our yeast from bunches of *V. vinifera* genotypes showing tolerance to *B. cinerea* because of the physical structure of the bunch generally associated with susceptibility to mold infections. These data can also represent a confirmation of our previous of co-evolution hypothesis of microorganisms within the growing area, to the extent of involving the genotype of the host plant [30]. Moreover, this hypothesis is supported also by other studies on *Arabidopsis* [69], maize [70], olive [71] and grapevine [72], suggesting a relationship between microbial communities in the phyllo-sphere and susceptibility to leaf pathogens. Exploiting this coevolution process could, in our opinion, represent a valid alternative strategy for a more rapid selection of microorganisms with antagonistic action.

Information on the mechanisms of action of the antagonists is essential to develop appropriate formulation and methods of application, to obtain registration and to select new effective microorganisms [16]. While competition for nutrients and space is considered being the primary mode of action of antagonistic yeasts against postharvest fungal pathogens [16], it is rare for just one mechanism of action to be involved in suppressing a disease alone. An effective biocontrol agent, in fact, is generally able to control a disease development by adopting several mechanisms of action that often work in concert. The in vitro tests performed in this work eventually confirmed the presence of different mecha-nisms of biocontrol for some of the selected yeast strains. *St. bacillaris* strain ‘N22_I1’ and *S. diversa* strain ‘N22_I3’ significantly inhibited the mycelium growth of the pathogen in the CALt assays, letting us hypothesize the production of fungistatic diffusible substances as a further mechanism of biocontrol action [30,46,73]. In addition, *S. diversa* strain ‘N22_I3’ as well as *A. pullulans* strain ‘OLB_9.1_VL’ significantly inhibited the mycelial growth of *B. cinerea* in the sandwich dual-culture assay [30,47,74], suggesting the production of VOCs as an inhibitory mechanism of action [75]. Finally, *St. bacillaris* S13_I6 and *H. uvarum* OLB_9_BR resulted unable to significantly reduce the in vitro growth of *B. cinerea*, suggesting a mechanism of action mainly by nutritional and spatial competition [76]. The effectiveness of our *A. pullulans* yeast strain in reducing the mycelial growth of *B. cinerea* by VOCs supported the previous evidence of combined mechanisms of this yeast in controlling the growth of the fungus [77,78]. Regarding *St. bacillaris*, many authors associate its ability to control the mycelial growth of *B. cinerea* with the production of VOCs [79,80], but the results obtained in this work indicated a different mechanism of action the *St. bacillaris* strain isolated for the experiments. This contrasting result suggests in our opinion the importance of further investigating the microbiome diversity, to identify BCAs with species-specific mechanisms of action. Results of the in vitro extracellular lytic enzymes assay supports the hypothesis regarding the mechanisms of action of selected yeasts. In fact, we observed that in the presence of the pathogen *B. cinerea*, *S. diversa* ‘N22_I3’ hydrolyzed lipids while *A. pullulans* ‘OLB_9.1_VL’ was able to hydro-lyse both lipids and proteins. Interestingly, this ability is lost when both yeast strains were cultivated in the absence of pathogens. Lipase and protease activity could explain the efficacy of these yeast strains to control *B. cinerea* in both in vitro and in vivo conditions as lipids and proteins are the main extracellular matrix (ECM) compounds produced by germlings of *B. cinerea* [81]. The ECM has several important roles in the infection process of the pathogen such as tropism towards the infection site, prevention of desiccation of conidia and matrix in which fungal toxins or enzymes required for the infection process are sequestered [82]. In addition, *A. pullulans* ‘OLB_9.1_VL’ expressed esterase activity when it was not in the presence of the pathogen, and this property could be a notable feature for the possible use of the selected yeast in winemaking, since esters commonly influence fruity aromas in wine [83]. Notably, isolate *St. bacillaris* ‘N22_I1’ did not show any of the tested enzymatic activities, therefore, additional investigations to further characterize their mechanism of action are needed.

To be considered a good BCA, a microorganism must possess some features, such as effectiveness at reasonable doses and against a wide range of pathogens, being able to survive in formulations easy to distribute and with long shelf-life and safe to human health [33]. The effectiveness of the yeast strains selected in this work was independent from their concentration of application. When in controlled conditions, they were actually effective against a wide range of pathogens that have simultaneously infected grapes in cold storage, with the exception of *A. pullulans* ‘OLB_9.1_VL’ which was unable to control *A. niger* infections. This ability is an important characteristic for yeasts to be considered good antagonists, because when applied at low concentrations, they could represent a benefit to eco-sustainable viticulture as they would minimize alterations of environmental, plant and soil equilibria, as would also provide an economic advantage in the commercial sector by targeting multiple pathogens simultaneously, hence reducing the use of different products to defeat multiple diseases.

A useful microbial formulation should be inexpensive to realize, easy to distribute to the specific environment and have a long shelf-life, preferentially also upon storage at elevated temperatures [84]. Freeze-drying or lyophilization is a three-step process that ideally ends up with a product that easily reverses back to its former structure upon rehydration [85]. Nevertheless, freeze-drying may negatively affect the vitality and physiological state of the yeast. This process can in fact induce mechanical damage due to the formation of ice crystals, eventually leading to cellular death during freezing [86]. Microbial survival in the lyophilization process depends on various factors such as the intrinsic resistance traits of the strain, density, physiological status of the microorganisms, and re-hydration conditions of the powder forms [87]. In this work, we evaluated the intrinsic resistance of selected yeast strains to lyophilization and the effect of storage temperature on the shelf-life of the obtained powder. While all the tested yeast strains showed intrinsic resistance to lyophilization (SFL = 1), the storage temperature significantly affected the shelf-life of the considered strains. Our data showed that the shelf-life of *St. bacillaris* ‘N22_I1’ and *S. diversa* ‘N22_I3’ was not affected by the storage temperature; otherwise, the room temperature significantly reduced the shelf-life of *A. pullulans* ‘OLB_9.1_VL’ and completely inhibited the vitality of *St. bacillaris* ‘S13_I6’ and *H. uvarum* ‘OLB_9_BR’. For these yeasts, further research is necessary to identify a lyoprotectant that can increase the shelf-life of freeze-dried products stored at high temperatures. In this context, previous work has demonstrated that the best way to formulate the biocontrol yeast *Wickerhamomyces anomalus* (E.C. Hansen) Kurtzman was the freeze-drying process, with the prior addition of trehalose to the yeast suspension. The authors demonstrated that the freeze-dried products could be stored at temperature as high as 30 °C for a year, with only a minor decrease in viability [84].

Human health risk assessment is a prerequisite for the application of a microorganism as Biological Control Agents [88]. In this work, we used erythrocytes to evaluate the potential toxicity of the studied antagonistic strains, which has previously been proposed as a useful biological model to study potential human health risks [89]. Our results showed that only *A. pullulans* ‘OLB_9.1_VL’ caused erythrocyte breakage (β-haemolysis) at 25 °C, therefore representing a risk for humans, raising concerns about its potential use as a biological control agent in agriculture.

In conclusion, we screened 31 different yeast strains, isolated from the carposphere of seven new table grape genotypes, to develop biological control alternatives against the grey mold of table grape. Based on in vivo assays, we proposed five non-*Saccharomyces* yeast strains as suitable BCAs against *B. cinerea*, labeled *St. bacillaris* ‘N22_I1’ and ‘S13_I6’, *S. diversa* ‘N22_I3’, *H. uvarum* ‘OLB_9_BR’ and *A. pullulans* ‘OLB_9.1_VL’. In contrast to the latter, the other five yeasts selected in the first in vivo experiment (‘N22_I4’, ‘N20_9B’, ‘AxAR4’, ‘CxM5’ and ‘OLB_6’) did not confirm their efficacy in the second experiment, a condition probably attributable to their genetic instability, an inherent characteristic of some wild yeast strains [83], which made them unsuitable for technological deepening as BCAs [30]. All the proposed yeast strains except for *A. pullulans* ‘OLB_9.1_VL’ showed a good predisposition for technological development thanks to (i) their efficiency at low concentrations and against a wide range of fungal pathogens; (ii) their ability to survive at the freeze-drying process; and (iii) their inability to cause haemolysis of red blood cells. Further research covering (i) evaluations both in field and postharvest conditions, (ii) biotechnological aspects of large-scale production and formulation, and (iii) identification of antifungal metabolites, are needed to develop bioproducts that can be used on biological and/or integrated control strategies for grey mold of table grapes.

## Figures and Tables

**Table 1:** Yeasts were tested on media in the presence of the pathogen (P.) and without the pathogen (W.P.) on plates. Isolates able to hydrolyze the compound are indicated by (+); those not able by (−); and isolates that did not grow on the specific substrate are indicated by (N.G.).

Yeast Strains	Extracellular Lytic Enzymes Activity											
	Lipase		Esterase		Β-1,3-Glucanase		Chitinase		Protease		Pectinase	
	P.	W.P.	P.	W.P.	P.	W.P.	P.	W.P.	P.	W.P.	P.	W.P.
*S. diversa* N22_I3	+	−	−	−	−	−	−	N.G.	−	−	−	−
*St. bacillaris* N22_I1	N.G.	N.G.	N.G.	N.G.	N.G.	N.G.	N.G.	N.G.	−	−	−	−
*A. pullulans* OLB_9.1_VL	+	−	−	+	−	−	−	−	+	−	−	−

**Table 2:** Yeast survival to the freeze-drying process and maintenance of their viability at +4 °C and +25 °C.

Yeast Isolate	SF_L_	SF_S_	
		+4 °C	+25 °C
*St. bacillaris* N22_I1	1.00 ± 0.0	0.85 ± 0.01	0.90 ± 0.02
*S. diversa* N22_I3	1.00 ± 0.0	0.70 ± 0.01	0.78 ± 0.01
*A. pullulans* OLB_9.1_VL	1.00 ± 0.0	0.67 ± 0.003	0.47 ± 0.003
*H. uvarum* OLB_9_BR	0.98 ± 0.01	0.00 ± 0.0	0.00 ± 0.0
*St. bacillaris* S13_I6	1.00 ± 0.0	0.78 ± 0.002	0.00 ± 0.0

**Table 3:** Effect of haemolytic action of the five yeasts plated on blood agar at 25 °C and 37 °C.

Yeast Isolate	25 °C	37 °C
*St. bacillaris* N22_I1	No growth	No growth
*S. diversa* N22_I3	γ-haemolysis	No growth
*St. bacillaris* S13_I6	No growth	No growth
*A. pullulans* OLB_9.1_VL	β-haemolysis	No growth
*H. uvarum* OLB_9_BR	No growth	No growth

## Data Availability

Raw sequencing data from Illumina sequencing experiments from this study have been submitted to the Sequence Read Archive (SRA; https://www.ncbi.nlm.nih.gov/sra/) under BioProject PRJNA1054966.

## Supplementary Materials

The following supporting information can be downloaded at: https://www.mdpi.com/article/10.3390/microorganisms12020340/s1, Table S1: New autochthonous genotypes of CREA-VE in Turi; Table S2. Molds classification; Figure S1. Pathogenicity tests; Table S3. Thirty-one new yeasts isolated from the grape berries of new seven autochthonous genotypes of CREA-VE in Turi; Table S4. Yeast characterization. Supplementary Material D.1.1. Sequencing of 5.8S_Internal Transcribed Spacer Region; Supplementary Material D.1.2. Sequencing of D1/D2 26S rDNA sequences.
