# Supplementary material for: On the Way to the Technological Development of Newly Selected Non-Saccharomyces Yeasts Selected as Innovative Biocontrol Agents in Table Grapes
Source: Microorganisms. 2024 Feb 6;12(2):340. doi: 10.3390/microorganisms12020340 (PMC10891982; doi:10.3390/microorganisms12020340)
Supplement: Supplementary file 1 [file microorganisms-12-00340-s001.zip › microorganisms-2833054-supplementary.pdf]

Title: "On the Way to the Technological Development of Newly Selected Non-*Saccharomyces* Yeasts Selected as Innovative Biocontrol Agents in Table Grapes"

Authors: Salerno Antonella[1,2], D'amico Margherita[1], Bergamini Carlo[1], Maggiolini Flavia Angela Maria[1], Vendemia Marco[1], Prencipe Annalisa[2], Claudia Rita Catacchio[2] Ventura Mario[2], Cardone Maria Francesca[1*] and Marsico Antonio Domenico[1*]

[1] Council for Agricultural Research and Economics, Research Center Viticulture and Enology (CREA-VE), Via Casamassima 148, 70010 Turi, Italy

[2] Department of Biosciences, Biotechnology and Environment, University of Bari "Aldo Moro", Via Orabona 4, 70125 Bari, Italy

[#] Correspondence: mariafrancesca.cardone@crea.gov.it; adomenico.marsico@crea.gov.it

Table S1: New autochthonous genotypes of CREA-VE in Turi. The table shows the codes of the seven grape genotype from which the isolation was carried out; the parental crossbreedings from which the genotypes derive; and the *B. cinerea* tolerance class to which the genotype belongs, according to OIV scale, sheet No. 459: 3=low, 5=medium, 9=high.

[*1] Pool of berries collected from tolerant genotypes that are part of the segregating population derived from the Almeria x Autumn Royal crossbreeding.

[*2] Pool of berries collected from tolerant genotypes that are part of the segregating population derived from the Cimminita B x Melissa B crossbreeding.

[*3] Pool of berries collected from tolerant genotypes that are part of the segregating population derived from the Red globe x Regal B crossbreeding.

[*4] Pool of berries collected from tolerant genotypes that are part of the segregating population derived from the Ceresa Rs x Carati B crossbreeding.

GENOTYPE	PARENTAL GENOTYPES	TOLERANCE CLASS	IMAGES
**AXAR*1**	Almeria<br>X<br>Autumn Royal	9	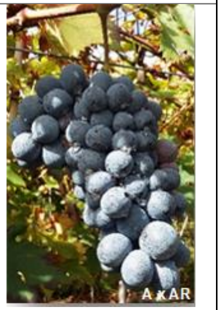
**CXM*2**	Cimminita B<br>X<br>Melissa B	9	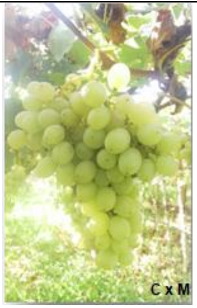
**N15/048*2**		3	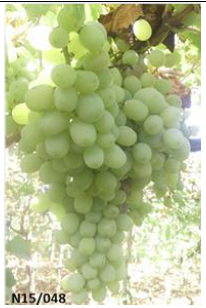

<table>
<tr><td>N20/057[*3]</td><td rowspan="2">Red globe
X
Regal B</td><td rowspan="2">9</td><td>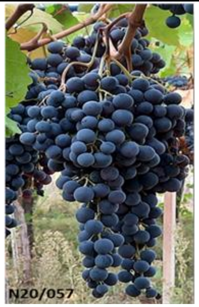</td></tr>
<tr><td>N22/132[*3]</td><td>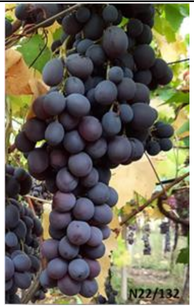</td></tr>
<tr><td>Olivetta B</td><td>Olivetta B</td><td>9</td><td>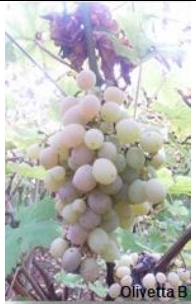</td></tr>
</table>

**S13/158**[*4]	Ceresa Rs<br>X<br>Carati B	5	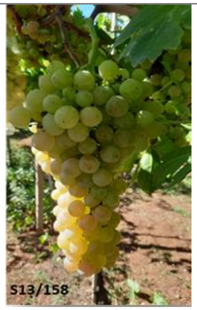

Table S2. Molds classification. Distinction of isolated molds by source matrix, taxonomic and morphologic classification. In the last column, the purified fungal colonies grown at 25°C on PDA medium.

**Fungal isolate code**	**Source of isolation**	**ITS Illumina sequencing match (% of hits)**[*]	**5.8S sequencing match (Sanger E-value/identity%)**	**Colony morphology**	**Images**
AS1	grape	*Botrytis cinerea* (94.12%)	/	White cottony mycelium in contact with the culture medium, with a gray filamentous mass on top	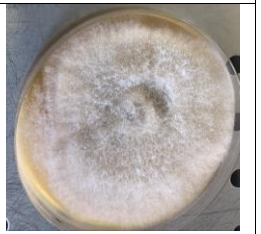
AS3	tomato	*Cladosporium spp.* (36.0%)	0.0/99.79	Olivaceous colony tending to brown, with velvety-dusty surface	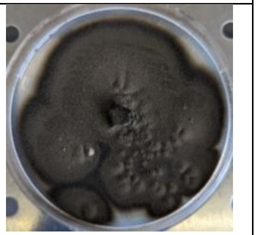

AS9	tangerine	*Alternaria alternata* (45.0%)	/	Dark green cottony mycelium with a white border in the growth zone, with a white filamentous mass on top	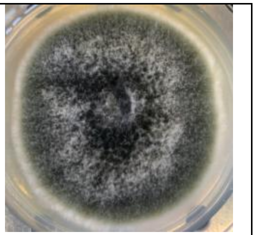
AS13	tangerine	*Penicillium digitatum* (94.2%)	/	Brownish-green powdery mycelium, with white borders in the growth zone	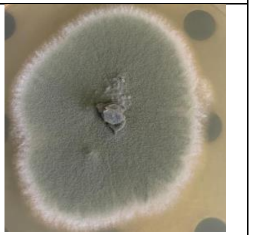
AS14	pomegranate	*Penicillium glabrum* (34.1%)	0.0/99.40	Greenish-blue powdery mycelium, with lighter borders in the growth zone	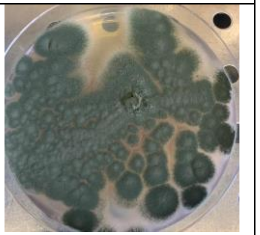
AS19	grape	*Aspergillus niger* (68.0%)	/	White colony covered by a dense mass of conidia with the aspect of small roundish brown-black granules	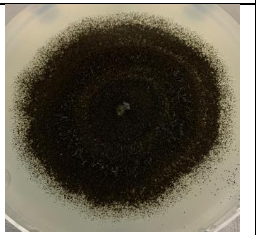

***% of matches to the closest related type strains calculated as the number of species-specific hits over the total number of hits identifying in GenBank database**

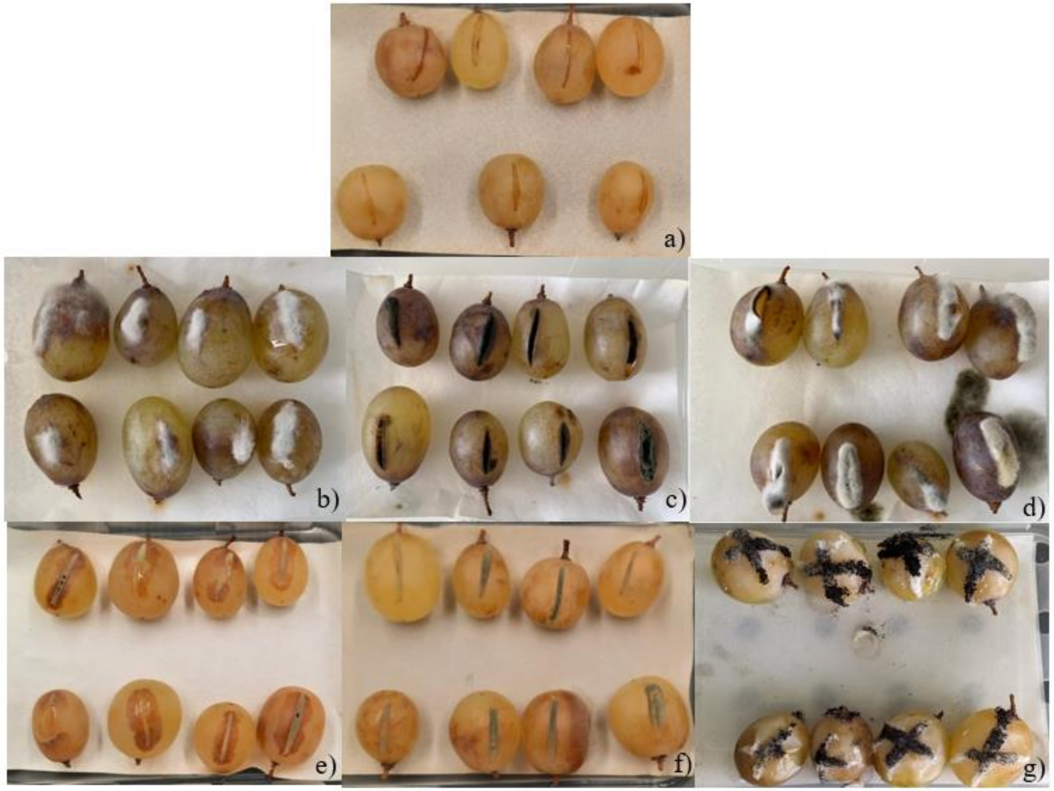

Figure S1. Pathogenicity tests. Grape berries infected with the six fungal pathogens, compared with the uninoculated control (a). All the berries show symptoms of the disease caused by: *Botrytis cinerea* b); *Cladosporium sp*. c); *Alternaria alternata* d); *Penicillium digitatum* e); *Penicillium glabrum* f); and *Aspergillus niger* g).

Table S3. Thirty-one new yeasts isolated from the grape berries of new seven autochthonous genotypes of CREA-VE in Turi.

	YEAST	COLONY COLOUR	COLONY TOPOGRAPHY	IMAGES
**1**	AXAR_1	Cream to light green/yellowish medium	Flat, surface: smooth/opaque	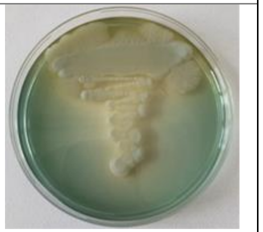
**2**	AXAR_1.1	Cream to light green/yellowish medium	Flat, surface: smooth/opaque	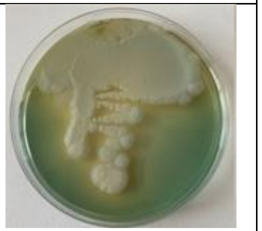
**3**	AXAR_1.2	White to cream/yellow medium	Convex, surface: wrinkled/opaque	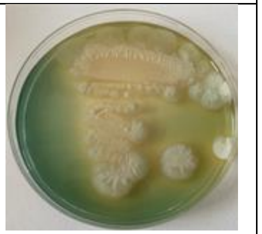
**4**	AXAR_4	Light blue in the center/cream at periphery	Flat, surface: smooth/glossy	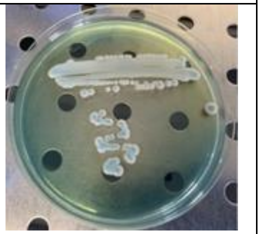

**5**	AXAR_5	White to cream/yellowish medium	Flat, surface: smooth/opaque	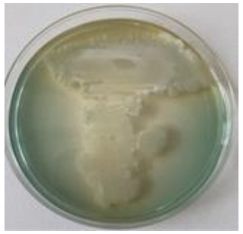
**6**	CXM2	Light blue in the center/cream at periphery	Flat, surface: smooth/glossy	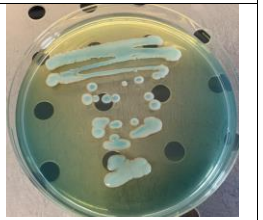
**7**	CXM3	Light green/yellowish medium	Flat, surface: smooth/glossy	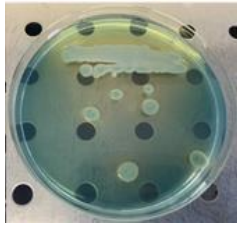
**8**	CXM5	Cream to light green/yellowish medium	Flat, surface: smooth/opaque	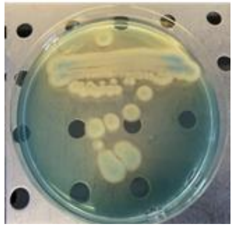
**9**	N15_E5	Intense green in the center/ cream at periphery	Flat, surface: smooth/glossy	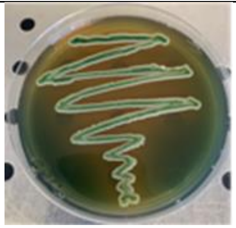

**10**	N20_9B	Light green with thin hyphal-like ramifications /yellowish medium	Convex, surface: smooth/glossy	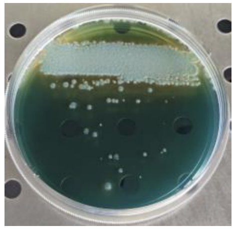
**11**	N20_9V	Light green with thin hyphal-like ramifications /yellowish medium	Convex, surface: smooth/glossy	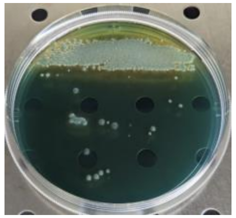
**12**	N20_I5	Dark green in the center/white at periphery	Flat, surface: smooth/glossy	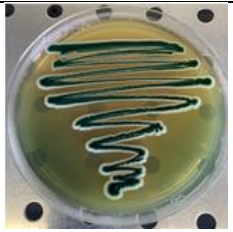
**13**	N22_E2	Intense green/greenish medium	Convex, surface: smooth/opaque	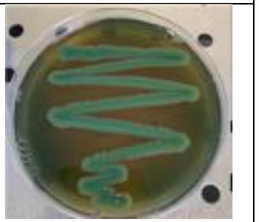
**14**	N22_E4	Intense green/greenish medium	Flat, surface: smooth/opaque	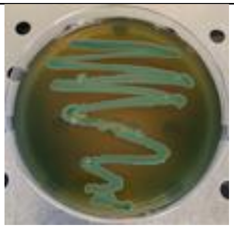

**15**	N22_E5	Intense green in the center/ cream at periphery	Flat, surface: smooth/glossy	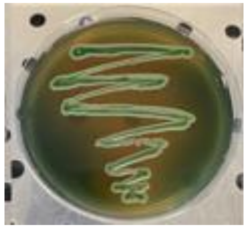
**16**	N22_I1	Intense green in the center/ white at periphery	Convex, surface: smooth/glossy	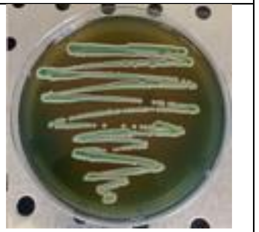
**17**	N22_I2	Intense green/greenish medium	Convex, surface: smooth/opaque	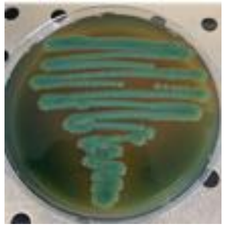
**18**	N22_I3	Light green/yellowish medium	Flat, surface: smooth/glossy	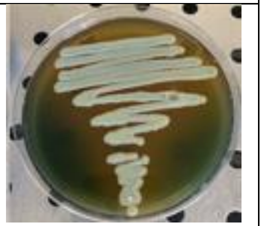
**19**	N22_I4	Intense green/ greenish medium	Flat, surface: smooth/opaque	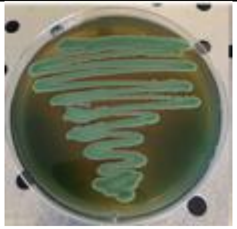

**20**	N22_I5	Intense green in the center/white at periphery	Flat, surface: smooth/glossy	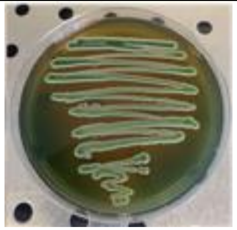
**21**	N22_I6B	Intense white in the center/light green at periphery	Convex, surface: smooth/glossy	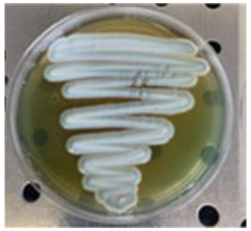
**22**	N22_I6V	Light green/ yellowish medium	Flat, surface: smooth/glossy	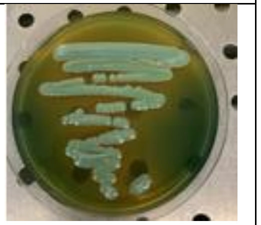
**23**	OLB_6	Light green/yellowish medium	Flat, surface: smooth/glossy	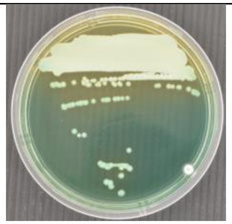
**24**	OLB_9.1_VL	Light green with thin hyphal-like ramifications /yellowish medium	Convex, surface: smooth/glossy	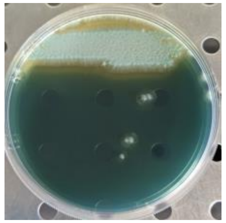

**25**	OLB_9_BR	Intense green in the center/ light green at periphery/ yellowish medium	Convex, surface: smooth/glossy	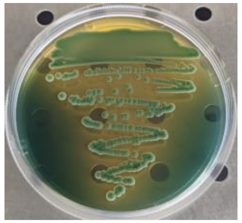
**26**	S13_I3	Intense green in the center/cream at periphery	Flat, surface: smooth/glossy	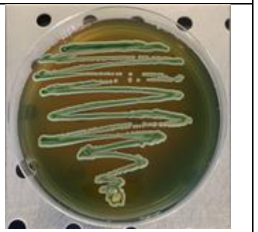
**27**	S13_I4	Light blue in the center/intense blue in the periphery	Convex, surface: smooth/opaque	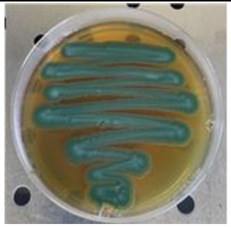
**28**	S13_I5	Dark green in the center/white at periphery	Convex, surface: smooth/glossy	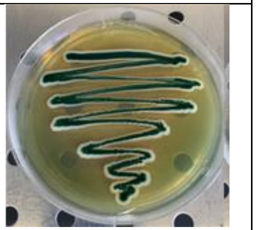

**29**	S13_I6	Dark green in the center/white at periphery	Convex, surface: smooth/glossy	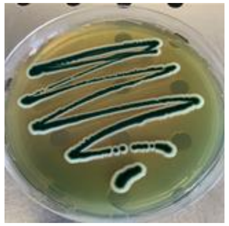
**30**	S13_I7	Light green tending to yellow/yellow medium	Flat, surface: smooth/opaque	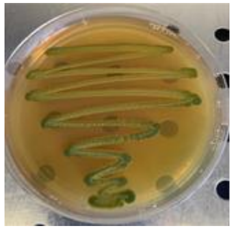
**31**	S13_I8	Intense green with light green edge/yellowish medium	Convex, surface: smooth/opaque	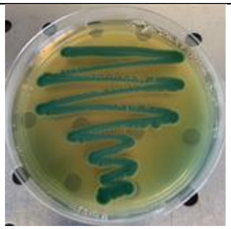

Table S4. Yeast characterization. The table shows the specific morphological characteristics of each yeast regarding colour and colony shape. Below the table are photographed plates of yeast colonies grown at 25°C on WL medium: a) N22_I1, b) N22_I3, c) S13_I6, d) OLB_9_BR, e) OLB_9.1_VL.

Yeast isolate code	ITS Illumina sequencing match		D1/D2 26S sequencing match (Sanger e-value/identity%)	Color (front view)	Topography	Images
	(% of hits) *	Best match (Accession/e-value/ identity%)				
N22_I1	*Starmerella bacillaris* (89.0%)	KT877395.1/ 3e-131/ 100 %	/	Intense green in the center with creamy-white border	Smooth, glossy, and shiny colony with convex surface	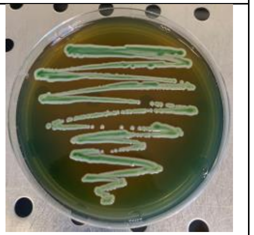
N22_I3	*Saturnispora diversa* (14.5%)	MN371911.1/ 9e-126/ 100 %	0.0/99.57	Cream tending to light green in the center	Smooth, glossy colony with flat surface, creamy aspect	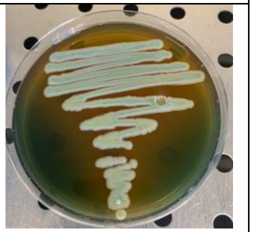
S13_I6	*Starmerella bacillaris* (89.0%)	KT877395.1/ 3e-131/ 100 %	/	Dark green in the center with white border	Colony with well-defined borders, glossy and shiny with convex surface	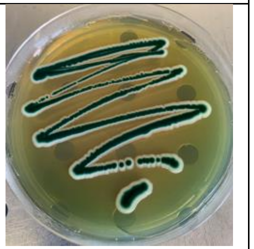
OLB_9_BR	*Hanseniaspora uvarum* (97.0%)	KT802750.1/ 4e-109/ 100%	/	Cream tending to light green	Wrinkled, buttery-looking colonies, condensed into small, dense glomeruli	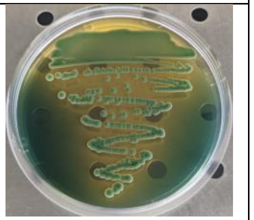

*% of matches to the closest related type strains calculated as the number of species-specific hits over the total number of hits identifying in GenBank database.

OLB_9.1_VL	*Aureobasidium pullulans* (70.0%)	KT722607.1/ 3e-141/ 100%	/	Intense blue	Small, rounded colonies denser and agglomerated in the center, with more transparent lateral radial streaks	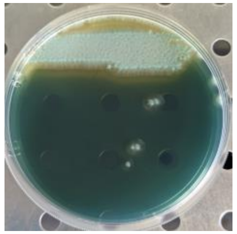

Supplementary material D1

D1.1 Sequencing of 5.8S_Internal Transcribed Spacer Region

>lcl|Query_29911:1-477 AS3_R.C12_22011910OI.scf (482 bp)*

CACCGGGATGTTCATAACCCTTTGTTGTCCGACTCTGTTGCCTCCGGGGCGACCCTGCCTTCGGGCGGGGGCTCCGGGTGGACACTTC
AAACTCTTGCGTAACTTTGCAGTCTGAGTAAACTTAATTAATAAATTAAAACTTTTAACAACGGATCTCTTGGTTCTGGCATCGATGAA
GAACGCAGCGAAATGCGATAAGTAATGTGAATTGCAGAATTCAGTGAATCATCGAATCTTTGAACGCACATTGCGCCCCCTGGTATTCC
GGGGGGCATGCCTGTTCGAGCGTCATTTCACCACTCAAGCCTCGCTTGGTATTGGGCAACGCGGTCCGCCGCGTGCCTCAAATCGACC
GGCTGGGTCTTCTGTCCCCTAAGCGTTGTGGAAACTATTCGCTAAAGGGTGTTCGGGAGGCTACGCCGTAAAACAACCCCATTTCTAA
GGTTGACCTCGGATCAGGTAGGCATACCCGCTGAA

* the alignment of the AS3 isolate's consensus sequence against the ITS fungal database showed the same percentage of identity (99.79%) with 10 species of the genus *Cladosporium*, so it was not possible to identify the species for this strain.

>lcl|Query_470369:1-500 AS14_R.F11_22011910OL.scf (500 bp)

ACCTGATCGAGGTCAACCTGATAAAAGGATGATTGGTTGTCGGCTGGCGCCGGCCGGGCCTACAGAGCGGGTGACAAAGCCCCATA
CGCTCGAGGACCGGACTCGGTGCCGCCGCTGCCTTTCGGACCCGTCCCCGGGGGGACGGAGCCCAACACACAAGCCGTGCTTGAGG
GCAGCAATGACGCTCGGACAGGCATGCCCCCCGGAATACCAGGGGGCGCAATGTGCGTTCAAAGACTCGATGATTCACTGAATTCT
GCAATTCACATTAGTTATCGCATTTCGCTGCGTTCTTCATCGATGCCGGAACCAAGAGATCCGTTGTTGAAAGTTTTAACTTATTTAG

TTTATGCTCAGACTGCAATCTTCAGACAGAGTTCAATAGTGTCTCCGGTGCGCGGACCCGGGGGCAGAAGCCCCCGGCGGCCGT
GAGGCGGGCGCACCGAAGCAACAAGGTACAATAAACACGGGTGGGAGGTTGGACCCAGAGGCCCTCA

D1.2 Sequencing of D1/D2 26S rDNA sequences

>Strain_N22_I3_NL1

TTGCCTCAGTAGCGGCGAGTGAAGCGGCAAAAGCTCCACTTTGAAAGCGTGTCGACGCGTTGTAGTGCGGTTCAGTCTTTGAGTGAC
GGATGACTAAGTCCCCTGGAACGGGGTGCCATAGAGGGTGAGAGCCCCGTGAGTTGTCTTTTTAGCTCTTTAAGTCTTTACCAAAGA
GTCGAGTTGTTTGGGAATGCAGCTCTAAGTGGGTGGTAAATTCCATCTAAGGCTAAATACCGGCGAGAGACCGATAGCGAACAAGT
ACAGTGATGGAAAGATGAAAAGCACTTTGAAAAGAGAGTGAAACAGTACGTGAAATTGTTGAAAGGGAAGGGTATTTGGCCCGAC
ATGGGTTCTGTGCACCGTTGCCTCTTGTAGGCGGCGCTCTGCTGGAGCCTGGGCCAGCATCAGTTTTCCGGCGAGGATAAGAAGTTT
TGAACCACATTGTNGGGGATGAGTTTGATGCTCGCA
